# Oral Fluids and Electrolytes in Non-diarrheal Diseases: A Cross-Sectional Study Assessing the Knowledge, Attitude, and Practices of Indian General Physicians

**DOI:** 10.7759/cureus.113499

**Published:** 2026-07-28

**Authors:** Sanjay Kalra, Chetan Patil, Amol Patil, Harshad Malve, Nikhil Bangale

**Affiliations:** 1 Endocrinology, Bharti Hospital, Karnal, IND; 2 Internal Medicine, Muktai Hospital, Nashik, IND; 3 Medical Affairs, Kenvue, Mumbai, IND; 4 Medical Safety Sciences, Kenvue, Mumbai, IND

**Keywords:** fluid electrolyte, hydration, india, non-diarrheal illness, recovery

## Abstract

Background and objective

Dehydration in non-diarrheal illnesses (NDIs) imposes significant fluid and electrolyte burdens, yet awareness and clinical practices regarding oral rehydration in this context remain insufficiently studied. This survey aimed to assess the knowledge, attitudes, and practices (KAPs) of general physicians (GPs) in India regarding oral fluid and electrolyte management in acute NDIs.

Methods

A cross-sectional online survey was conducted among verified GPs across India using a validated, four-domain questionnaire covering participant demographics, knowledge, attitudes, and clinical practices. The survey was completed by 1,333 GPs (MBBS: 48.5%; AYUSH (Ayurveda, Yoga, and Naturopathy, Unani, Siddha and Homoeopathy): 51.5%). Data were analyzed descriptively as frequencies (percentages) and numbers of responses or GPs.

Results

Fever/febrile illness was the most recognized cause of dehydration in NDIs (24.6%), with elderly patients identified as most vulnerable (23.4%). Most GPs (89.9%) acknowledged that fluid-electrolyte deficits impair immune function, and 87% agreed dehydration delays recovery by approximately 5.1 days. Attitudes toward ready-to-drink (RTD) isotonic electrolyte solutions were highly favorable: 92.6% considered them superior to plain water, 91.7% believed they accelerate recovery, and 92% agreed that formulations containing calcium, magnesium, and taurine support faster recovery in NDIs. Palatability emerged as a key compliance driver: 89.6% rated taste as very important or important, 84.9% endorsed lemon-flavored drinks for improving acceptance and countering nausea, and 83.3% agreed sucralose enhances palatability within safe permissible limits. In practice, RTD solutions (32.7%) were frequently recommended, as they shortened the GPs’ self-reported perception of recovery by 5.2 days, though only 60.9% of GPs routinely assessed hydration status.

Conclusions

GPs demonstrate broadly favorable KAP toward RTD isotonic electrolyte use in NDIs. Gaps in dehydration recognition and routine assessment highlight the need for targeted physician education and evidence-based clinical guidelines.

## Introduction

Dehydration assessment presents significant clinical difficulties due to its complex pathophysiology, ambiguous clinical manifestations, and the absence of global consensus on its definition and diagnosis. Despite this complexity, both clinical practice and medical literature tend to frame dehydration almost exclusively within the context of diarrheal illness. Dehydration may result from either inadequate water intake or excessive water loss, with causes including fever, vomiting, pulmonary hyperventilation, scalds, and diabetes. These reflect a broader etiological spectrum that is frequently under-represented in standard rehydration guidance, which continues to treat gastroenteritis as the near-exclusive indication for oral rehydration therapy.

Febrile illnesses, persistent vomiting, heat-related conditions, and acute viral gastroenteritis are all common in the tropical and subtropical climate of India and impose significant fluid and electrolyte burdens, requiring prompt oral or parenteral correction [1-15]. A retrospective real-world analysis of electronic medical records (2017-2023) estimated that non-diarrheal illnesses (NDIs) accounted for approximately 18.5 million cases in India. Although dehydration and electrolyte imbalance frequently accompany these conditions, they remain substantially underrecognized and underdocumented, with a recorded prevalence of 0.026% (n = 4,917) among patients with NDIs. This finding highlights potential gaps in clinical recognition, assessment, and documentation [16]. Similarly, older adults are particularly susceptible to dehydration, as polypharmacy, reduced thirst perception, and renal senescence increase the physiological rationale for timely oral rehydration [17].

Commercially available oral ready-to-drink (RTD) beverages are commonly referred to as hydration drinks or electrolyte drinks and are formulated to replenish body fluids lost during dehydration. These hydration or electrolyte drinks contain salts such as sodium chloride, sodium citrate, and potassium chloride, reflecting their essential role in restoring the body's fluid and electrolyte balance and facilitating hydration during periods of dehydration [5,18,19]. However, structured guidance and clinical evidence for hydration management using oral fluid-electrolyte solutions in NDIs remain limited [1,5,18,20,21]. Factors such as palatability, gastrointestinal tolerance, and caloric considerations can influence the suitability of rehydration strategies to meet broader clinical and patient-centric needs in acute NDIs [1,16,18,19,22]. Moreover, electrolyte imbalance may not be adequately corrected by plain water alone, especially in anorexic patients with poor oral intake [1].

Oral fluid-electrolyte solutions, particularly RTD formulations, contain balanced electrolytes (such as sodium, potassium, and magnesium), nutrients (such as taurine), carbohydrate (glucose), and low-sugar non-nutritive sweeteners (such as sucralose). Hence, they offer a practical approach to such non-diarrheal scenarios by providing balanced hydration and electrolyte replenishment, which are critical determinants of fluid absorption, without the need for reconstitution from powdered formulation [5,18,19]. The electrolytes contained within these drinks, principally sodium, potassium, and associated mineral salts, are collectively referred to as hydration salts, reflecting their essential role in restoring the body's fluid and electrolyte balance during states of dehydration.

The awareness, perceptions, and clinical practices related to dehydration and the use of fluid-electrolyte solutions in NDIs among general physicians (GPs) remain insufficiently explored [23,24]. To address this evidence deficit, the present cross-sectional survey was designed to assess the knowledge, attitudes, and practices (KAPs) of general physicians in India regarding the use of oral fluid-electrolyte solutions in non-diarrheal conditions, to inform targeted educational interventions and guideline development.

## Materials and methods

Design and administration of the questionnaire

A cross-sectional online survey questionnaire was developed to evaluate the KAP of GPs regarding their approach to the management of dehydration with fluid and electrolyte imbalance in patients with NDIs across India. The questionnaire consisted of four domains. The first domain covered the profiles of the participating GPs. The second domain assessed knowledge of the causes and clinical manifestations of dehydration in patients with acute NDIs, as well as the identification of populations at increased risk. It also evaluated awareness of the effects of fluid and electrolyte imbalances on immune function, knowledge of the optimal composition (macro- and micronutrients) of electrolyte drinks, and understanding the role of isotonic RTD formulations in the effective management and recovery of dehydration. The third domain determined GPs' attitudes toward dehydration in NDIs, including its clinical impact, monitoring needs, and perceptions on the effectiveness, safety, and suitability of RTD electrolyte formulations across settings. The fourth domain evaluated the real-world clinical practices in managing dehydration in acute NDIs, including assessment patterns, treatment approaches, prescribing behaviors, and decision-making factors guiding the selection and use of oral hydration strategies, particularly RTD electrolyte drinks in routine care.

The questionnaire was intended primarily as a descriptive and exploratory tool rather than a psychometric scale; formal validation procedures such as construct validity testing or factor analysis were not undertaken. To ensure content relevance and clarity, the questionnaire was developed following a review of the relevant literature and discussion by a panel of experts, and was evaluated by subject-matter experts for content appropriateness and comprehensiveness.

This survey was conducted among qualified GPs (MBBS and AYUSH (Ayurveda, Yoga and Naturopathy, Unani, Siddha, and Homoeopathy) practitioners) practicing across India. GPs were selected using a convenience sampling approach. Eligible participants included qualified GPs currently engaged in active clinical practice and managing patients with NDIs, who provided informed consent before participation and whose responses remained anonymous. Physicians in non-clinical roles (e.g., administrative or academic-only positions) and those who did not provide informed consent or submitted incomplete questionnaire responses were excluded from the analysis.

The questionnaire was administered using an online platform (SurveyMonkey) via a secure link. Data collection was carried out over a period of 10 days, from April 9 to April 18, 2026. Of the 10,000 GPs invited to participate, 1,333 completed the questionnaire, corresponding to a response rate of 13.33%. Results were obtained as frequencies (percentages) and the number of responses or GPs. All responses were securely captured and stored within the SurveyMonkey platform. For questions permitting multiple responses, participants could select more than one option; therefore, the denominators varied across questions. Percentages were calculated based on the total number of responses received for each option using Microsoft Excel. Access to the data was restricted to authorized personnel only. The collected data, including responses, were securely stored (with restricted access) and analyzed.

Sample size calculation

The sample size was calculated using the Wilson score confidence interval (CI) method for a single proportion in SAS (PROC POWER), which estimates the required sample size through an iterative numerical algorithm based on the Wilson score confidence interval approach [25]. The corresponding formula is presented below:

(p̂ + z²/2n ± z√[p̂(1-p̂)/n + z²/4n²]) / (1 + z²/n)

Assuming a proportion of 30% (*p̂* = 0.3), a two-sided 95% confidence level (*z*-score corresponding to the desired confidence level, 1.96), and a precision (half-width) of 5.4%, a sample size (*n*) of 288 provides approximately an 87% probability of achieving the desired precision. After accounting for a 10% non-response rate, the final sample size (*n_1_*) was inflated to 320 GPs, as shown below.

n_1_= n/ (1 - r)

where *n_1_* is the adjusted sample size, *n* is the calculated sample size (288), and *r* is the anticipated non-response rate (0.10).

Ethical consideration

The participation of GPs in the study was voluntary after adequate information was provided to them. Participants reviewed and accepted the informed consent form before proceeding with the questionnaire response. This survey was conducted in accordance with the Declaration of Helsinki. Ethics approval (KAP EC Reference No.- ICH/EC/2026/003) was obtained from the Infinity Care Hospital Ethics Committee, Varanasi, dated April 7, 2026.

## Results

A total of 1,453 GPs took the survey, of which 1,333 GPs (response rate: 13.3%) completed the survey questionnaire. Participants were evenly distributed across specialties (MBBS: 48.5%; AYUSH: 51.5%). Respondents represented a wide geographical distribution across multiple states of India (refer to the footnote of Table 1), encompassing diverse levels of experience and clinical practice settings (Table 1). When responses were stratified by specialties, MBBS and AYUSH practitioners demonstrated comparable response patterns across knowledge, attitude, and practices domains.

**Table 1:** Participant demographics Geographical distribution of the participants: Andhra Pradesh (10.6%), Arunachal Pradesh (0.1%), Assam (3.3%), Bihar (6.4%), Chandigarh (0.1%), Chhattisgarh (0.9%), Delhi (2.5%), Gujarat (1.2%), Jammu and Kashmir (0.4%), Jharkhand (3.1%), Karnataka (4.8%), Kerala (0.6%), Madhya Pradesh (5%), Maharashtra (14%), Manipur (0.4%), Meghalaya (0.2%), Mizoram (0.2%), Nagaland (0.1%), Odisha (4.6%), Pondicherry (0.1%), Punjab (0.7%), Rajasthan (3.4%), Tamil Nadu (7.3%), Telangana (8.5%), Tripura (0.7%), Uttarakhand (0.6%), Uttar Pradesh (9.7%), West Bengal (10.3%), and not specified (0.1%) AYUSH: Ayurveda, Yoga, and Naturopathy, Unani, Siddha, and Homoeopathy

Characteristics	N (%)
Specialty	
MBBS	48.5 (647)
AYUSH	51.5 (686)
Years of experience	
< 5	11.6 (154)
5-10	31.9 (426)
10-20	30.9 (412)
> 20	25.6 (341)
Type of practice	
Private	76.4 (1,019)
Hospital-based	9.1 (122)
Both	14.4 (192)
Average number of patients seen per day	
< 20	27.4 (366)
20-40	54.5 (727)
41-60	13.6 (182)
> 60	4.3 (58)

Knowledge

Understanding the Importance of Dehydration in Illness

**Figure 1:**
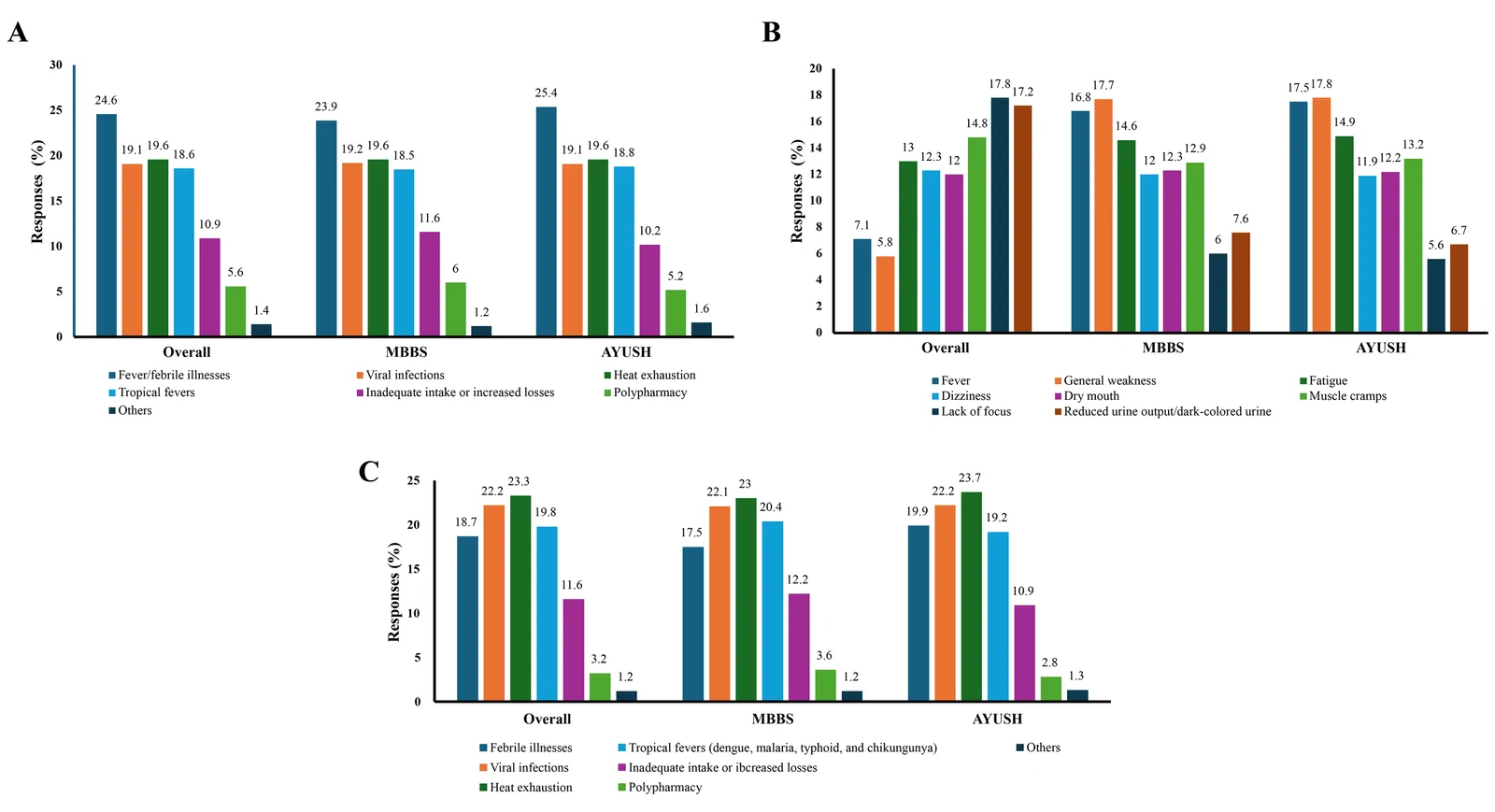
Understanding dehydration in acute illnesses (A) Conditions leading to dehydration in NDIs Multiple responses were permitted, and percentages are based on the total number of responses (N = 4,418 (overall), 2,172 (MBBS), 2,246 (AYUSH)). Others: excessive sweating, diabetes and related conditions (polyuria or ketoacidosis), nausea, vomiting, exercise without replacement, alcohol intake, burns, gastrointestinal disorders, iron deficiency anemia, diarrhea, endocrine disorders (including hyperthyroidism), burns, anti diabetics (SGLT2 inhibitors), diuretics (B) Symptoms associated with dehydration Multiple responses were permitted, and percentages are based on the total number of responses (N = 6,026 (overall), 2,921 (MBBS), 3,105 (AYUSH)) (C) Physicians’ perception for recommendation of RTD electrolyte drinks Multiple responses were permitted, and percentages are based on the total number of responses (N = 4,177 (overall), 2,060 (MBBS), 2,117 (AYUSH)). Others: tropical fevers, sunstroke, vomiting, nausea, diabetes, general weakness, burns, gastrointestinal conditions, loose motion, orthostatic hypotension and tachycardia, dry mucous membranes, reduced salivation, decreased urine output and dark-colored urine AYUSH: Ayurveda, Yoga, and Naturopathy, Unani, Siddha, and Homoeopathy; NDI: non-diarrheal illness; SGLT2: sodium/glucose cotransporter 2; RTD: ready-to-drink

Among the overall cohort, fever/febrile illnesses were the most frequently cited conditions (24.6%, n = 1,089) by the GPs as leading causes of dehydration in NDIs, followed by heat exhaustion (19.6%, n = 867), viral infections (19.1%, n = 846), and tropical fevers, including dengue, malaria, typhoid, and chikungunya (18.6%, n = 824; total responses: N = 4,418; Figure 1A). Similarly, general weakness (17.8%, n = 1,072), fever (17.2%, n = 1,036), and fatigue (14.8%, n = 890) were considered the key symptoms associated with dehydration in acute illnesses (total responses: N = 6,029; Figure 1B). The most common indications for recommending RTD electrolyte drinks among physicians were heat exhaustion (23.3%, n = 975) and viral infections (22.2%, n = 926; total responses: N = 4,177; Figure 1C).

Among patient populations, the elderly (23.4%, n = 1,115) were considered the most susceptible to dehydration or electrolyte deficits, followed by pediatric patients (15.6%). In comparison, patients on polypharmacy (3.9%, n = 187) and those in palliative care (3.4%, n = 160) were considered the least susceptible (total responses: N = 4,757). Key contributing factors to fluid and electrolyte deficits among the elderly included reduced total body water (32.4%, n = 984) and multiple comorbidities (23.6%, n = 718), while impaired thirst sensation accounted for only 18.6% (n = 567) of the 3,041 total responses. In contrast, awareness was higher for specific clinical associations: 88.5% (n = 1,180) of GPs agreed that heavy alcohol consumption can lead to dehydration, and 88.8% (n = 1,184) recognized that patients undergoing chemotherapy or radiotherapy remain susceptible to fluid and electrolyte deficits despite IV fluid administration (Table 2).

**Table 2:** Physicians’ knowledge of dehydration in acute illnesses and their management across specialties ^*^Multiple responses were allowed, and percentages are based on the total number of responses to the respective statement (total responses: N = (A) 4,757 (overall), 2,349 (MBBS), 2,399 (AYUSH); (B) 3,041 (overall), 1,497 (MBBS), 1,544 (AYUSH)) Data represented as % (n) AYUSH: Ayurveda, Yoga, and Naturopathy, Unani, Siddha, and Homoeopathy

	Overall	MBBS	AYUSH
A. Patient populations susceptible to dehydration or electrolyte deficits^*^			
Pediatric	15.6 (743)	15.1 (356)	15.8 (378)
Elderly	23.4 (1115)	22.9 (540)	23.9 (575)
Pregnant	6.3 (301)	6.1 (144)	6.5 (157)
Individuals fasting	10.3 (489)	10.3 (243)	10.2 (246)
People involved in strenuous physical activity	9.2 (438)	8.8 (208)	9.6 (230)
People with acute infections	11.6 (553)	11.5 (271)	11.7 (282)
Patients with diabetes	11.2 (535)	11.5 (270)	11 (265)
Individuals with obesity	5 (236)	5.1 (121)	4.8 (115)
Patients on polypharmacy	3.9 (187)	4.3 (102)	3.5 (85)
Patients in palliative care	3.4 (160)	4 (94)	2.7 (66)
B. Elderly patients are more susceptible to dehydration due to^*^			
Less total body water	32.3 (984)	30.5 (457)	34.1 (527)
Reduced antidiuretic hormone	18.8 (571)	18.2 (272)	19.4 (299)
Impaired thirst sensation	18.6 (567)	19 (284)	18.3 (283)
Multiple comorbidities	23.6 (718)	24.9 (373)	22.3 (345)
Polypharmacy	6.6 (201)	7.4 (201)	5.8 (111)

Perceptions on Immune Function, Electrolyte Composition, and Impact of Dehydration on Recovery

The majority (89.9%, n = 1,199) of GPs affirmed that fluid and electrolyte deficits impact immune function in pediatric and elderly populations and in patients with lifestyle disturbances. The GPs believed that sodium (23.7%, n = 1,233), potassium (22.2%, n = 1,156), and chloride (20.8%, n = 1,079) were the most essential electrolytes in electrolyte drinks, followed by magnesium (15.0%, n = 781), calcium (13.0%, n = 675), bicarbonate/citrate (4.6%, n = 241), and others (including taurine, vitamin B, vitamin C, glucose, dextrose, zinc, selenium, and L-carnitine; 0.6%, n = 30; total responses: N = 5,195). Among the nutrients assessed for their perceived role in immune support, zinc was most frequently identified (17.6%, n = 1,034), followed by selenium (13.3%, n = 785), sodium (12.5%, n = 737), potassium (12.3%, n = 727), vitamin E (12.1%, n = 712), magnesium (11.5%, n = 675), chloride (11.0%, n = 650), and calcium (9.2%, n = 544; total responses: N = 5,887).

**Figure 2:**
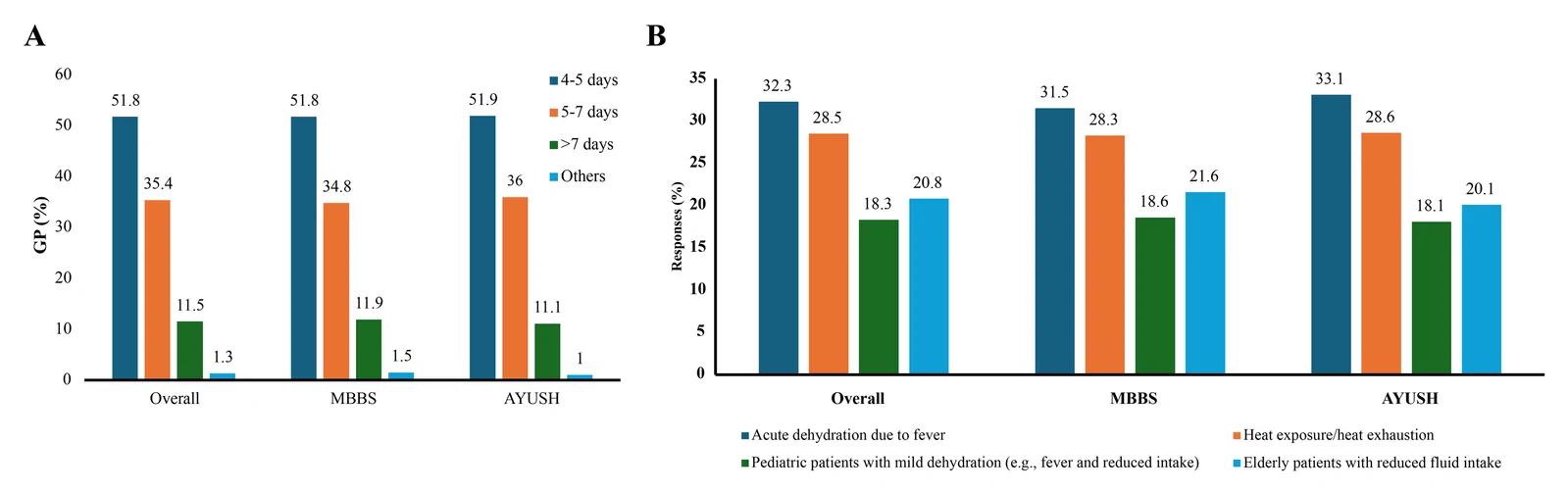
Effect of dehydration on patient recovery and its management (A) In acute NDIs, dehydration can delay recovery by: others: 1-2 days, 2-3 days, ≥ 3 days, 14 days with ORS and zinc, and depends on patient condition and severity of dehydration (B) Physicians’ perception toward recommendation of RTD electrolyte drinks. Multiple responses were allowed for the question, and percentages are based on the total number of responses (N = 3,401 (overall), 1,647 (MBBS), 1,754 (AYUSH)) GP: general physician; AYUSH: Ayurveda, Yoga, and Naturopathy, Unani, Siddha, and Homoeopathy; NDI: non-diarrheal illness; ORS: oral rehydration solution; RTD: ready-to-drink

Additionally, 90.5% (n = 1,207) of GPs agreed that dehydration during acute NDIs can delay patient recovery and negatively impact the quality of life of patients until recovery. A majority (87%, n = 1,163) of GPs believed that dehydration delays recovery by four to seven days (weighted average: 5.1 days) (Figure 2A). A substantial proportion of GPs (86%, n = 1,147) agreed that isotonic fluids enhance fluid absorption compared with plain water or hypertonic solutions. Most GPs (78.5%, n = 1,047) reported that isotonic fluids are unlikely to exacerbate diarrhea even if consumed during an active diarrheal episode. The majority of GPs agreed that RTD electrolyte drinks speed recovery in acute NDIs (91.7%, n = 1,223), provide faster hydration than plain water (92.6%, n = 1,234), are more reliable than homemade beverages (90.4%, n = 1,205), reduce fatigue or weakness (93.3%, n = 1,243), and are recommended for febrile tropical illnesses (92.6%, n = 1,234), everyday dehydration scenarios (89.5%, n = 1,193), post-dental procedures (71.5%, n = 953), and postoperative dehydration, including spinal headaches (73.2%, n = 976).

Electrolyte content was identified as the primary determinant of effective hydration (39.6%, n = 1,050), followed by osmolality/isotonicity (25.1%, n = 667), sugar concentration (17.9%, n = 474), and taste and palatability (17.4%, n = 461; total responses: N = 2,562). The GPs considered acute dehydration due to fever (32.3%, n = 1,100) to be the leading indication for recommending RTD electrolyte drinks, followed by heat exposure or heat exhaustion (28.5%, n = 968), elderly patients with reduced fluid intake (20.8%, n = 709), and pediatric patients with mild dehydration, such as those with fever and reduced intake (18.3%, n = 624; total responses: N = 3,401) (Figure 2B).

Attitudes

Perspective on Hydration Practices and Use of Electrolyte Drinks

**Figure 3:**
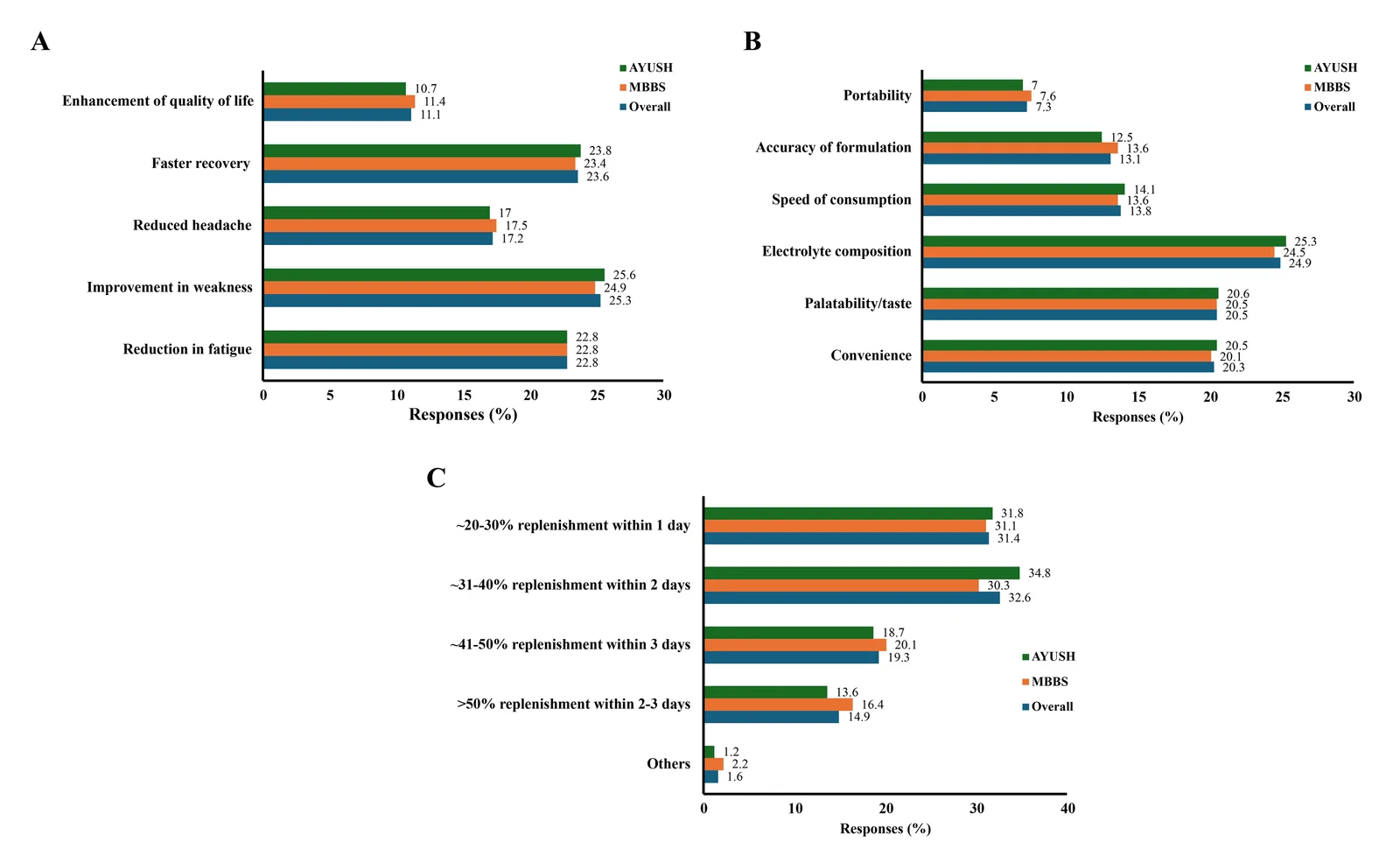
Attitude of the general physicians towards the management of dehydration in NDIs using RTD electrolyte drinks (A) Clinical benefits of RTD electrolyte drinks. Multiple responses were permitted, and percentages are based on the total number of responses (N = 3,903 (overall), 1,901 (MBBS), 2,002 (AYUSH)) (B) Factors affecting suitability of RTD electrolyte solutions. Multiple responses were permitted, and percentages are based on the total number of responses (N = 3,746 (overall), 1,860 (MBBS), 1,886 (AYUSH)) (C) Fluid–electrolyte replenishment AYUSH: Ayurveda, Yoga, and Naturopathy, Unani, Siddha, and Homoeopathy; NDI: non-diarrheal illness; RTD: ready-to-drink

The majority (90.5%, n = 1,207) of the GPs agreed that dehydration in acute NDIs delays recovery and worsens quality of life. Among the GPs, 92.6% (n = 1,234) regarded RTD electrolyte drinks as a more effective hydration strategy compared to plain water, while 90.4% (n = 1,205) believed that RTD electrolyte drinks were more reliable than homemade beverages such as salt-sugar solutions and lemon water. In addition, most of the GPs (91.7%, n = 1,223) considered RTD electrolyte drinks to speed recovery in acute NDIs (Table 3). RTD electrolyte drinks were believed to accelerate overall recovery in NDIs (23.6%, n = 922), alleviate multiple dehydration-related symptoms, including fatigue (22.8%, n = 890), weakness (25.3%, n = 986), and headache (17.2%, n = 673), thereby enhancing quality of life (11.1%, n = 432; total responses: N = 3,903; Figure 3A).

**Table 3:** Attitude of the GPs on dehydration in acute NDIs and their management across specialties ^*^Sucralose is a permitted non-nutritive sweetener under the Food Safety and Standards (Food Products Standards and Food Additives) Regulations, 2011, allowing use in ready-to-serve beverages up to 300 ppm (300 mg/L). The Joint FAO/WHO Expert Committee on Food Additives (JECFA) and the European Food Safety Authority (EFSA) have confirmed that sucralose is acceptable for general food use and have established an Acceptable Daily Intake (ADI) of 0-15 mg/kg body weight Data represented as % (n) GP: general physician; NDI: non-diarrheal illness; AYUSH: Ayurveda, Yoga, and Naturopathy, Unani, Siddha, and Homoeopathy; RTD: ready-to-drink

	Overall	MBBS	AYUSH
Dehydration in acute NDIs can delay recovery and negatively impact the quality of life of patients until recovery			
Agree	90.5 (1,207)	90.2 (584)	90.8 (623)
Neutral	7.8 (104)	8.2 (53)	7.4 (51)
Disagree	1.6 (22)	1.5 (10)	1.7 (12)
While WHO oral rehydration solution is the treatment of choice for diarrheal dehydration, it is recommended to use RTD isotonic electrolyte drinks to address dehydration in acute non-diarrheal illnesses)			
Agree	86 (1,146)	84.2 (545)	87.6 (601)
Neutral	10.2 (136)	11.3 (73)	9.2 (63)
Disagree	3.8 (51)	4.5 (29)	3.2 (22)
RTD electrolyte drinks fasten recovery in acute NDIs			
Agree	91.7 (1,223)	91.5 (592)	92 (631)
Neutral	7 (93)	7.3 (47)	6.7 (46)
Disagree	1.3 (17)	1.2 (8)	1.3 (9)
RTD electrolyte drinks provide faster hydration compared to plain water alone			
Agree	92.6 (1,234)	91.2 (590)	93.8 (644)
Neutral	6.1 (81)	6.9 (45)	5.2 (36)
Disagree	1.3 (18)	1.8 (12)	0.9 (6)
RTD electrolyte solutions are more reliable than homemade beverages (e.g., salt–sugar solution and lemon water) for managing dehydration in acute NDIs			
Agree	90.4 (1,205)	89.6 (580)	91.1 (625)
Neutral	7.5 (100)	7.3 (47)	7.7 (53)
Disagree	2.1 (28)	3.1 (20)	1.2 (8)
RTD electrolyte drinks help reduce fatigue or weakness associated with dehydration			
Agree	93.3 (1,243)	92.4 (598)	94 (645)
Neutral	6.1 (82)	7 (45)	5.4 (37)
Disagree	0.6 (8)	0.6 (4)	0.6 (4)
RTD electrolyte drinks are recommended for optimal hydration in febrile illnesses like tropical fevers (dengue, malaria, typhoid, and chikungunya)			
Agree	92.6 (1,234)	90.6 (586)	94.5 (648)
Neutral	6.4 (86)	8.5 (55)	4.5 (31)
Disagree	1 (13)	0.9 (6)	1 (7)
RTD electrolyte drinks help address symptoms associated with dehydration in everyday depletion or dehydration scenarios, such as exhaustion, physical or mental stress, exercise, or brain fog (lack of focus)			
Agree	89.5 (1,193)	87.9 (569)	90.9 (624)
Neutral	8.5 (114)	9.6 (62)	7.6 (52)
Disagree	1.9 (26)	2.5 (16)	1.4 (10)
RTD electrolyte drinks are recommended to address fluid and electrolyte deficits after dental procedures such as root canals, dental extractions, or periodontal surgeries			
Yes	71.5 (953)	71.7 (464)	71.3 (489)
No	12.2 (163)	11.3 (73)	13.1 (90)
Not sure	16.3 (217)	17 (110)	15.6 (107)
The use of electrolyte drinks is considered appropriate for managing dehydration in postoperative patients, including those with spinal headaches			
Yes	73.2 (976)	72.6 (470)	73.8 (506)
No	10.1 (135)	9.7 (63)	10.5 (72)
Not sure	16.6 (222)	17.6 (114)	15.7 (108)
When used within permissible limits, sucralose^* ^enhances palatability, demonstrating a wide margin of safety and tolerability across diverse populations			
Agree	83.3 (1,111)	81.3 (526)	85.3 (585)
Neutral	14.5 (194)	15.6 (101)	13.6 (93)
Disagree	2.1 (28)	3.1 (20)	1.2 (8)
What is your perception of sucralose as a sweetener in ready-to-drink electrolyte drinks?			
Safe and acceptable	52.7 (703)	50.1 (324)	55.2 (379)
Acceptable with caution	29.9 (399)	28.6 (185)	31.2 (214)
Neutral	14 (187)	16.4 (106)	11.8 (81)
Not preferred	3.3 (44)	4.9 (32)	1.8 (12)

The GPs expressed favorable attitudes toward the use of RTD electrolyte drinks for specific health conditions, including fatigue or weakness (93.3%, n = 1,243), febrile illnesses (including tropical fevers such as dengue, malaria, typhoid, and chikungunya; 92.6%, n = 1,234), everyday depletion or dehydration scenarios (including exhaustion, stress, exercise, and cognitive fatigue; 89.5%, n = 1,193), after dental procedures (71.4%, n = 953), and for postoperative dehydration support (including spinal headaches; 73.2%, n = 976; Table 3). A substantial proportion of GPs believed that formulations containing calcium, magnesium, and taurine support faster recovery in conditions such as heat exhaustion and viral illness-related myalgia (92%, n = 1,233), as well as menstrual cramps (81.4%, n = 1,085).

Electrolyte balance (24.9%, n = 933), palatability (20.5%, n = 770), and convenience (20.3%, n = 761) were identified as the key drivers for recommending RTD electrolyte drinks (total responses: N = 3,746; Figure 3B). The majority (32.6%, n = 435) of the GPs believed that RTD isotonic electrolyte drinks provide approximately 31 to 40% replenishment of fluid and electrolytes in patients with acute dehydration due to NDIs within two days (Figure 3C). Notably, 91.1% (n = 1,214) agreed that low-sugar isotonic formulations are appropriate for managing non-diarrheal dehydration, while 77.7% (n = 1,035) considered lemon-flavored isotonic electrolyte drinks to be well tolerated in pregnancy-related nausea and morning sickness. The GPs perceived that restoration of fluid balance and hydration (39.3%, n = 957), electrolyte correction (36.8%, n = 896), and improved glucose availability (23.1%, n = 562) were the primary mechanisms underlying the relief of hangover symptoms such as headache, dizziness, and brain fog following alcohol consumption (total responses: N = 2,435). Attitudes toward sweeteners were favorable, with most GPs (83.3%, n = 1,111) agreeing that sucralose enhances palatability while maintaining safety and tolerability within permissible limits, and it was considered either a safe (52.7%, n = 703) or an acceptable sweetener with caution (29.9%, n = 399; Table 3).

Practices

**Figure 4:**
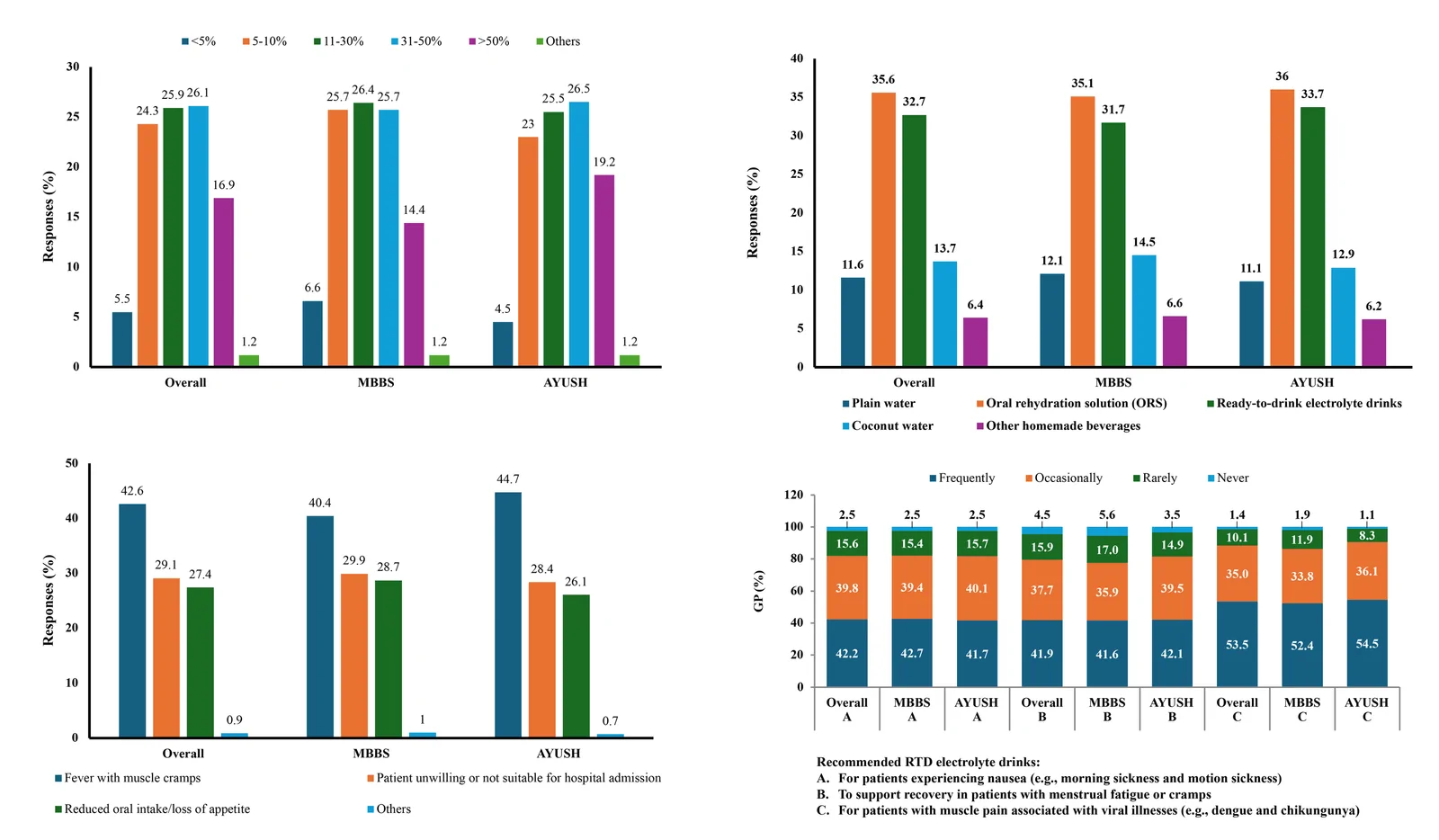
Clinical practice in dehydration risk and its management in NDIs (A) Patient population at risk of dehydration (B) Management of dehydration. Multiple responses were permitted, and percentages are based on the total number of responses (N = 2,741 (overall), 1,361 (MBBS), 1,380 (AYUSH)) (C) Symptoms and conditions associated with recommendation of RTD electrolyte drinks. Multiple responses were permitted, and percentages are based on the total number of responses (N = 2,524 (overall), 1,259 (MBBS), 1,265 (AYUSH)). Others: excess heat exposure, vomiting, and nausea (D) Frequency of recommendation of RTD electrolyte drinks NDI: non-diarrheal illness; AYUSH: Ayurveda, Yoga, and Naturopathy, Unani, Siddha, and Homoeopathy; GP: general physician; RTD: ready-to-drink

Across specialties, most GPs reported that they performed routine evaluation of hydration status in patients presenting with acute NDIs. Most of the GPs (60.9%, n = 812) indicated that they always or often assessed hydration. While 20.6% (n = 275) and 16.6% (n = 221) of GPs reported that they evaluated hydration status occasionally and rarely, respectively. The majority (76.3%, n = 1,018) of GPs perceived that, in their practice, nearly half of the patients (5-50%) with NDIs are at risk of dehydration, necessitating fluid-electrolyte replenishment (Figure 4A). They believed that oral rehydration solution (ORS) (35.6%, n = 975) and RTD electrolyte solutions (32.7%, n = 896) were the most frequently recommended hydration solutions (total responses: N = 2,741; Figure 4B).

Among GPs, 54.3% (n = 724) noted that they recommended RTD electrolyte drinks as adjuvants to support hydration and recovery to at least half of their febrile patients. Meanwhile, 29.2% (n = 390) indicated that they extended this recommendation to three-quarters of patients, and 15.4% (n = 205) reported recommending them to all febrile patients. A large majority of GPs (91.3%, n = 1,218) supported the use of RTD isotonic electrolyte drinks as adjuvant therapy in all patients with febrile illnesses to enhance hydration and recovery. The GPs described fever accompanied by muscle cramps (42.6%, n = 1,075) as the leading cause prompting the recommendation of RTD electrolyte drinks (total responses: N = 2,524; Figure 4C). They also reported frequently recommending these electrolyte drinks for patients experiencing nausea (42.2%, n = 562), menstrual fatigue or cramps (41.9%, n = 558), and muscle pain associated with viral illnesses (53.5%, n = 713; Figure 4D).

**Figure 5:**
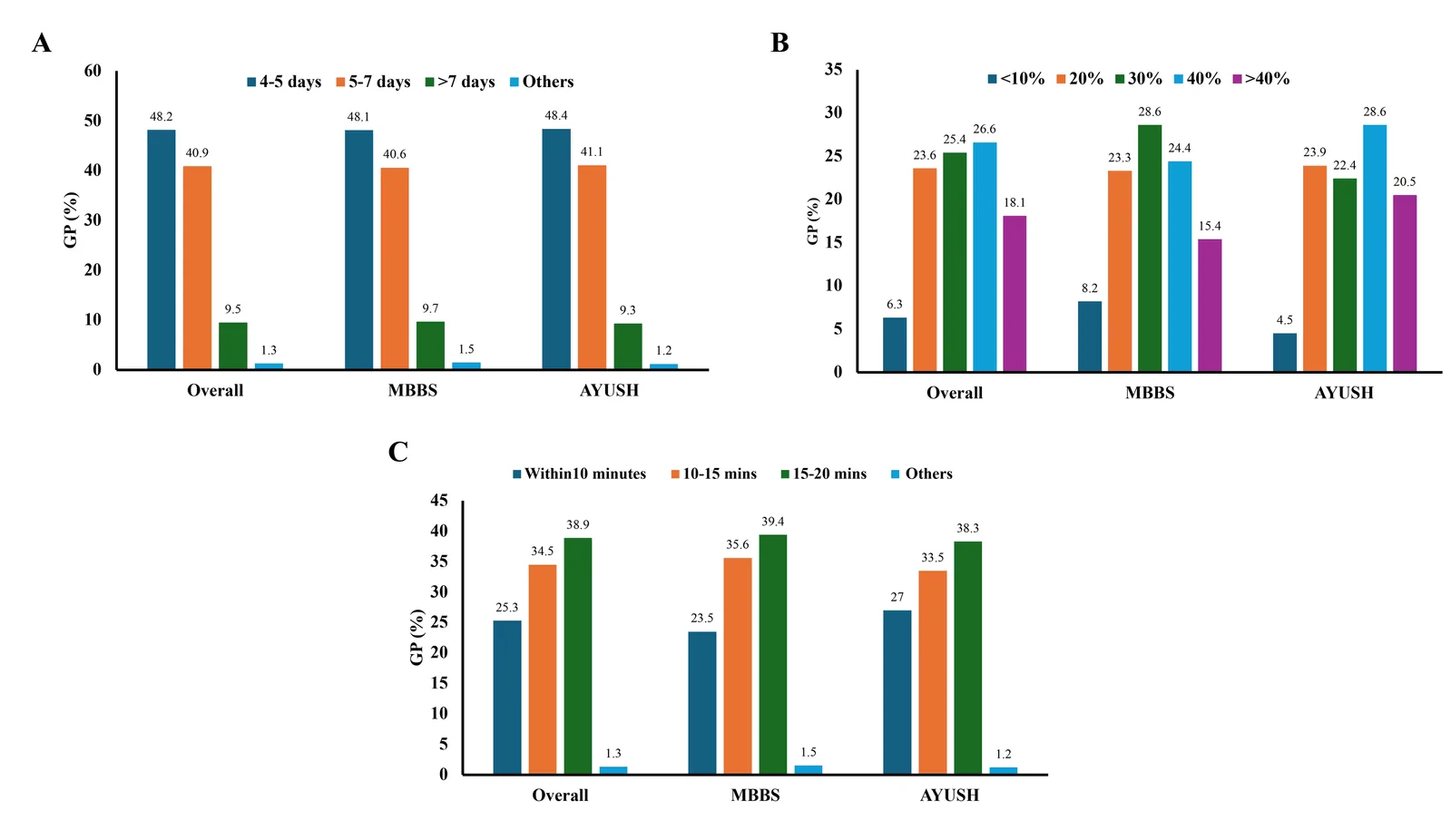
Clinical utilization of RTD electrolyte formulations in rehydration (A) Speed of recovery. Others: 1-2 days, 2-3 days, 14 days, not sure, did not use or specify (B) Extent of rehydration using RTD electrolyte drinks (C) Rehydration outcome and time to recovery with RTD electrolyte drinks. Others: ≥ 30 min, 30-45 minutes, 40-50 minutes, 1-2 hours, not sure, did not use, and specify RTD: ready-to-drink; GP: general physician; AYUSH: Ayurveda, Yoga, and Naturopathy, Unani, Siddha, and Homoeopathy

In their clinical practice, the majority of GPs (89.1%, n = 1,188) believed that RTD electrolyte drinks helped to shorten/accelerate recovery duration by four to seven days (weighted average: 5.2 days; for illnesses typically lasting approximately 15 days), while 75.6% (n = 1,008) perceived a 20-40% improvement in the speed of recovery (Figures 5A, 5B). Among GPs, 98.7% (n = 1,315) reported that they believed RTD electrolyte drinks help patients with NDIs feel hydrated within 20 minutes of consumption (Figure 5C).

In the present survey, most GPs (86.5%, n = 1,153) preferred RTD isotonic drinks, depending on the electrolyte formulation, sugar level, and palatability (Table 4). In addition, 90% (n = 1,199) of GPs considered RTD isotonic, low-sugar electrolyte solutions to be a suitable option for managing dehydration in acute NDIs (including diabetes and obesity), irrespective of comorbidities and concomitant medications. In patients receiving antibiotics, 93.5% of GPs (n = 1,247) reported recommending RTD electrolyte formulations containing prebiotics to help address antibiotic-associated gut dysbiosis. GPs also indicated a willingness to recommend adjuvant treatments containing prebiotics (22.1%, n = 295), probiotics (16.5%, n = 220), or both (60.1%, n = 801) to address antibiotic-associated gut dysbiosis (Table 4).

**Table 4:** Attributes of RTD electrolyte drinks Data represented as % (n). Others: choosing a suitable antibiotic with less propensity for gut dysbiosis or stopping the antibiotic altogether; depends on patient condition RTD: ready-to-drink; AYUSH: Ayurveda, Yoga, and Naturopathy, Unani, Siddha, and Homoeopathy; NDI: non-diarrheal illness

	Overall	MBBS	AYUSH
Preference for type of electrolyte drink for your patients with acute NDIs			
Isotonic	86.5 (1153)	85 (550)	87.9 (603)
Hypertonic	9.7 (129)	10.1 (65)	9.3 (64)
Hypotonic	3.8 (51)	4.9 (32)	2.8 (19)
Importance of taste in ensuring patient compliance with an oral hydration drink			
Very important	56 (747)	51.8 (335)	60.1 (412)
Important	33.6 (448)	37.2 (241)	30.2 (207)
Moderately important	8.8 (118)	9.1 (59)	8.6 (9.1)
Not important	1.5 (20)	1.8 (8)	1.2 (8)
Preferred level of sugar in oral electrolyte drinks			
Low sugar	59.5 (793)	58.3 (377)	60.6 (416)
Moderate sugar	24.7 (330)	23.8 (154)	25.7 (176)
Sugar-free	13.4 (179)	15 (97)	11.9 (82)
Does not make a difference	2.3 (31)	2.9 (19)	1.8 (12)
In your experience, do lemon-flavored electrolyte drinks improve patient acceptance and help counter nausea?			
Agree	84.9 (1132)	82.5 (534)	87.2 (598)
Neutral	13.3 (177)	16 (103)	10.8 (74)
Disagree	1.8 (24)	1.5 (10)	2.0 (14)
Ideal composition (electrolytes, sugar level, and additional nutrients) of RTD electrolyte drink for patients with acute illness			
Na, K, and Cl with zero sugar	19.3 (257)	21.6 (140)	17.1 (117)
Na, K, and Cl with optimal sugar for hydration	42.8 (571)	40.7 (263)	44.9 (308)
Na, K, and Cl with high sugar	3.3 (44)	4.3 (28)	2.3 (16)
Na, K, Cl, Ca, and Mg with optimal sugar to support muscle function	34.6 (461)	33.4 (216)	35.7 (245)
Recommendation of RTD electrolyte formulation containing prebiotics to patients receiving antibiotics			
Yes, routinely	30.7 (409)	28.8(186)	32.5 (223)
Yes, in selected patients	42.8 (570)	44.2(286)	41.4 (284)
Occasionally, depending on the clinical condition	20.1 (268)	20.1 (130)	20.1 (138)
No, I would not consider it	2.7 (36)	3.2 (21)	2.2 (15)
Not sure	3.8 (50)	3.7 (24)	3.8 (26)
Preferred adjuvant treatment to address antibiotic-associated dysbiosis			
Prebiotics alone	22.1 (295)	20.2 (131)	23.9 (164)
Probiotics alone	16.5 (220)	18.4 (119)	14.7 (101)
Both prebiotics and postbiotics	60.1 (801)	59.3 (384)	60.8 (417)
Others	1.3 (17)	2 (13)	0.6 (4)

## Discussion

This large-scale, cross-sectional survey of 1,333 GPs, encompassing both MBBS and AYUSH practitioners across diverse geographic regions and practice settings in India, demonstrated a broadly favourable KAP profile toward the management of dehydration in acute NDIs using RTD isotonic electrolyte drinks, while also identifying critical knowledge gaps and practice inconsistencies that warrant targeted interventions. The response patterns between the two specialties, MBBS and AYUSH, were broadly comparable across knowledge, attitude, and practice domains, including similar agreement rates on dehydration-related recovery delays, RTD electrolyte preferences, and clinical indications.

Fever was the most recognized cause of dehydration in NDIs (24.6%), consistent with prior evidence indicating that maintenance fluid requirements rise by approximately 12% for every degree of body temperature above 37.5°C [1]. However, dehydration markers, such as reduced urine output (7.1%) and brain fog (5.8%), were markedly underrecognized, indicating the need for structured training in dehydration assessment. This finding is in line with a multidisciplinary consensus, which indicated that subjective symptoms are poor indicators of dehydration, particularly in the elderly, and recommended a composite clinical assessment [26]. The recognition of heat exhaustion and tropical infections (dengue, malaria, typhoid, and chikungunya) as leading causes also aligns with existing reports, including national guidelines and expert recommendations, which emphasized fluid-electrolyte replenishment as central to supportive care in these conditions [3-5,9,18].

Elderly patients were recognized as the most vulnerable population (23.4%), which was attributed to numerous factors, including reduced total body water and multiple comorbidities. Similarly, earlier studies have indicated the multifactorial susceptibility of older adults to dehydration, including age-related reductions in total body water, diminished thirst sensation, impaired renal concentrating ability, polypharmacy, and comorbidity burden [27,28]. Notably, the underappreciation of impaired thirst, as endorsed by only 18.6% of GPs, is concerning because it is a key driver of inadequate voluntary fluid intake in the elderly and may lead clinicians to underestimate fluid deficits in the absence of overt thirst complaints [27,28].

Another study demonstrated that older individuals show blunted thirst responses even under conditions of significant water deprivation, making routine clinical assessment of hydration status essential, independent of patient-reported thirst [29]. Despite high awareness of dehydration risk among patients undergoing chemotherapy or radiotherapy, GPs infrequently identified those in palliative care (3.4%) or on polypharmacy (3.9%) as at-risk populations. This contrasts with prior evidence highlighting their elevated susceptibility to fluid-electrolyte deficits due to homeostatic alterations, anorexia, nausea, and poor oral intake, even alongside concurrent intravenous fluid administration, suggesting a gap that may translate to suboptimal hydration support in these groups [5,18].

The GPs strongly agreed that fluid and electrolyte deficits adversely impact immune function, with nutrients such as zinc, selenium, vitamin E, sodium, potassium, magnesium, chloride, and calcium being endorsed as supportive of immune function. Consistent with these findings, previous studies have associated habitual low water intake with increased free radicals and reduced antioxidant capacity, and have established the critical role of micronutrients, including zinc, selenium, and vitamin E, in supporting immune function and acting as antioxidants in addition to hydration [30,31]. Another study demonstrated that water restriction disrupted gut homeostasis, promoting microbial overgrowth and reducing colonic immune cells, particularly Th17 cells, thereby impairing pathogen clearance [32].

Our research highlighted that physicians perceived that dehydration in acute NDIs may delay recovery by 5.1 days, corroborating a real-world observational study in the Indian pediatric population demonstrating the role of oral electrolytes in shortening recovery durations in NDIs [33]. The present findings reinforce the understanding that RTD electrolyte solutions, widely recognized as hydration drinks or electrolyte drinks, remain central to the correction of dehydration when formulated according to standardized electrolyte compositions. The GPs perceived that isotonic RTD electrolyte solutions promote faster hydration and efficient fluid absorption compared to plain water and homemade beverages. These isotonic RTD formulations, with osmolality similar to plasma, contain balanced concentrations of electrolytes, including sodium, and carbohydrates that facilitate efficient fluid absorption via the sodium-glucose cotransporter mechanism and restoration of hydration [5,34,35]. A randomized, double-blind, placebo-controlled crossover trial involving healthy male workers exposed to occupational heat demonstrated that consumption of an electrolyte beverage containing electrolytes (sodium, potassium, and calcium) and carbohydrates was more effective in preventing dehydration than a non-electrolyte beverage [36].

Another randomized clinical trial assessing the impact of isotonic beverages in healthy Chinese adults demonstrated that their carbohydrate and electrolyte contents enhanced arginine vasopressin-mediated renal water reabsorption and improved fluid retention compared with plain water [37]. This highlights the importance of distinguishing properly formulated hydration drinks, containing hydration salts such as sodium chloride, sodium citrate, and potassium chloride in clinically validated proportions, from common household beverages that lack an equivalent electrolyte profile. GPs demonstrated a positive attitude toward RTD electrolyte drinks across a range of clinical scenarios, including fatigue or weakness (93.3%), febrile tropical illnesses (92.6%), menstrual cramps (81.4%), postoperative dehydration (73.2%), and post-dental procedure recovery (71.4%), suggesting a broadening of the conceptualization of hydration therapy beyond diarrheal contexts. This is in line with recent expert panel recommendations from India that advocated for oral fluid-electrolyte management across a wider spectrum of clinical presentations for NDIs, including elderly and pediatric care, transition care, and post-hospitalization recovery [1,5,18].

The high agreement (92%) that formulations containing calcium, magnesium, and taurine support faster recovery in conditions such as heat exhaustion and viral myalgia reflects physician recognition of the multidimensional role of electrolytes in neuromuscular function [15]. In the context of palatability, 89.6% of GPs rated taste as very important or important for patient compliance, and 84.9% agreed that lemon-flavored electrolyte drinks improve acceptance and help counter nausea. These attitudes are substantiated by clinical evidence demonstrating that sucralose-sweetened oral rehydration solutions are significantly more palatable than rice-based alternatives, and that palatability is a key determinant of intake volume and adherence [38]. In the present survey, 83.3% of GPs agreed that sucralose enhances palatability while maintaining safety within permissible limits, consistent with internationally established safety profiles for sucralose at permissible dietary doses endorsed by the European Food Safety Authority (EFSA) and WHO [39,40]. A randomized clinical trial also reported that non-nutritive sweeteners, including sucralose, had no significant effect on glycemic control [41].

Despite high awareness, a notable practice gap was identified: 60.9% of GPs reported always or often assessing hydration status in NDI patients, while 16.6% assessed it only rarely. This discordance between knowledge and practice is consistent with findings from earlier KAP surveys among Indian physicians on oral fluid management in NDIs, which documented gaps in the assessment and management of hydration [23,24]. ORS (35.6%) and RTD electrolyte solutions (32.7%) were the most frequently recommended hydration solutions for NDIs. Despite similar preferences between conventional ORS and RTD solutions, reflecting ongoing ambiguity regarding the optimal formulation, GPs perceived that WHO-ORS can be used for rehydration in patients with NDIs; however, concerns related to palatability, electrolyte composition, and osmolality may influence its suitability and patient adherence in certain clinical contexts [19]. A real-world, retrospective database study of adults and older adults in India demonstrated a high prevalence of dehydration among patients with NDIs, with fewer patients than expected receiving structured oral electrolyte therapy [22].

Our study also demonstrated that GPs indicated a growing trend toward the use of RTD electrolyte formulations in primary care, as evident from the observation that 64.8% of GPs reported recommending RTD electrolyte drinks to at least half of their febrile patients, and 91.3% supported their use as adjuvant therapy in febrile illnesses. The GPs noted that RTD electrolyte solutions may shorten recovery duration by 5.2 days for illnesses typically lasting approximately 15 days. Other reported clinical observations included 75.6% of GPs reporting a 20-40% improvement in recovery speed, and 73.4% of GPs documenting patient-reported hydration within 10-20 minutes of RTD electrolyte consumption [42].

Strengths and limitations

This study has several notable strengths. The large sample size (N = 1,333 completed responses) provides adequate statistical power and robust representativeness across Indian geographic regions, experience levels, and practice types. The inclusion of both MBBS (48.5%) and AYUSH (51.5%) practitioners in nearly equal proportion represents a more holistic view of primary care delivery in India, especially where AYUSH practitioners constitute a substantial proportion of the frontline healthcare workforce, particularly in rural and semi-urban settings. Furthermore, it focuses on dehydration associated with non-diarrheal illnesses, a clinically important yet underexplored area, which has received comparatively limited attention in current clinical research and practice. In addition, the multi-domain questionnaire design, spanning critical and unexplored domains of KAPs, allows for a nuanced assessment of the clinical landscape beyond simple awareness metrics. However, certain limitations must be acknowledged. First, as an online survey, the study was subject to selection bias, as participation was limited to GPs with digital access and willingness to engage with voluntary surveys. Second, as the study relied on self-reported responses, the findings may be subject to a degree of recall bias, which is an inherent limitation common to survey-based research.

## Conclusions

This survey demonstrates that Indian GPs across both MBBS and AYUSH specialties demonstrate positive knowledge and perceptions toward dehydration management in acute NDIs using RTD isotonic electrolyte solutions. However, some gaps remain in recognizing key dehydration markers, the vulnerability of elderly patients, and the hydration needs of patients in palliative care and those receiving polypharmacy, alongside inconsistent recommendation practices. These findings highlight the need for evidence-based clinical guidelines, structured physician education targeting identified knowledge gaps, and rigorous randomized controlled trials to establish the efficacy of RTD electrolyte formulations within Indian healthcare settings.
